# Comparison between the influence of roxadustat and recombinant human erythropoietin treatment on blood pressure and cardio-cerebrovascular complications in patients undergoing peritoneal dialysis

**DOI:** 10.3389/fmed.2023.1166024

**Published:** 2023-06-08

**Authors:** Shuiqin Cheng, Tingting Zhou, Le Yu, Zhihong Zhang, Yunmin Chen, Man Zhang, Jingjing Cui, Wenxin Yu, Jian Zhou, Yusheng Yu

**Affiliations:** Department of Nephrology, National Clinical Research Center of Kidney Disease, Jinling Hosptial, Nanjing University School of Medicine, Nanjing, China

**Keywords:** roxadustat, recombinant human erythropoietin, peritoneal dialysis, blood pressure, cardio-cerebrovascular complications

## Abstract

**Introduction:**

Roxadustat treatment in PD patients is equivalent to ESAs in increasing hemoglobin (Hb). But blood pressure, cardiovascular parameters, cardio-cerebrovascular complications and prognosis in the two groups before and after treatment has not been sufficiently discussed.

**Methods:**

Sixty PD patients who were treated with roxadustat for renal anemia in our PD center recruited from June 2019 to April 2020 as roxadustat group. PD patients treated with rHuEPO were enrolled at a 1:1 ratio as rHuEPO group using the method of propensity score matching. Hb, blood pressure, cardiovascular parameters, cardio-cerebrovascular complications and prognosis were compared between the two group. All patients were followed up for at least 24 months.

**Results:**

There were no significant differences in baseline clinical data or laboratory values between roxadustat group and rHuEPO group. After 24 months of follow-up, there was no significant difference in Hb levels (*p > 0.05*). There were no significant changes in blood pressure, or the incidence of nocturnal hypertension before and after treatment in roxadustat group (*p > 0.05*), while blood pressure significantly increased in rHuEPO group after treatment (*p < 0.05*). Compared with roxadustat group after follow-up, rHuEPO group had a higher incidence of hypertension, the levels of cardiovascular parameters were worse and cardio-cerebrovascular complications had a higher incidence (*p < 0.05*). Cox regression analysis showed age, systolic blood pressure, fasting blood glucose, and rHuEPO use before baseline were risk factors for cardio-cerebrovascular complications in PD patients, while treatment with roxadustat was a protective factor for cardiovascular and cerebrovascular complications.

**Conclusion:**

Compared with rHuEPO, roxadustat had less influence on blood pressure or cardiovascular parameters, and it was associated with a lower risk of cardio-cerebrovascular complications in patients undergoing PD. Roxadustat has a cardio-cerebrovascular protective advantage in PD patients with renal anemia.

## Introduction

Treatment with erythropoiesis-stimulating agents (ESAs) is an important therapy in peritoneal dialysis (PD) patients with renal anemia (1). When high doses of ESAs were used to raise serum hemoglobin (Hb) and hematocrit to achieve target values, the risk of cardio-cerebrovascular events and death increased (2, 3). Roxadustat is a hypoxia-inducing factor prolyl hydroxylase inhibitor (HIF-PHI), hypoxia-inducing factor (HIF) is a transcription factor that acts as the body’s primary oxygen partial pressure sensor, coordinating red blood cell production and Hb response. Roxadustat can be used for the treatment of renal anemia in patients with chronic kidney disease had good effectiveness and safety (4–6).

Recent studies (7, 8) have shown that roxadustat treatment in PD patients is equivalent to ESAs in increasing Hb. A previous study (9) found that compared with ESAs, roxadustat had no significant difference in blood pressure or cardiovascular effects in patients with renal anemia in chronic kidney disease (CKD); however, these comparisons were rarely reported in PD patients.

In this study, we retrospectively compared the effect of roxadustat and recombinant human erythropoietin (rHuEPO) on blood pressure, cardiovascular parameters and cardio-cerebrovascular complications in PD patients with renal anemia.

## Material and method

### Study design

This retrospective study analyzed renal anemia patients who underwent PD from June 2019 to April 2020 in the PD center of the National Clinical Research Center of Kidney Diseases in China. We enrolled 60 PD patients who were treated with roxadustat via oral administration for renal anemia as roxadustat group and 60 PD patients who were treated with rHuEPO via subcutaneous injection in a 1:1 ratio as rHuEPO group. The two groups were matched 1:1 by clinical data and biochemical data using the method of propensity score matching. Follow-up was finished in March 2022. All patients were followed up for at least 24 months.

### Inclusion and exclusion criteria

The inclusion criteria were as follows: (1) 18–80 years old, (2) PD patients with renal anemia, and (3) the treatment with roxadustat or rHuEPO was not interrupted during follow-up.

The exclusion criteria were as follows: (1) history of cardiovascular and cerebrovascular diseases, (2) abnormal liver function, (3) anemia due to hematological system diseases, tumors, rheumatoid diseases, gastrointestinal hemorrhage, and so on, (4) the treatment with roxadustat or rHuEPO was interrupted during follow-up, (5) incomplete clinical data.

### Treatment strategy

The initial dose of roxadustat in PD patients weighing between 45 and 60 kg was 100 mg; in patients weighing ≥60 kg, the initial dose was 120 mg three times a week according to the instructions. The follow-up dose of roxadustat was adjusted to keep the latter target Hb level. rHuEPO was used according to the degree of anemia, age, initial dose and dose adjustment based on package insert guidelines. The Hb target is 110 ~ 120 g/dL, but no more than 130 g/dL. When transferrin saturation (TSAT) ≤20% or (and) ferritin ≤100 μg/L, oral iron therapy was the first choice. Iron status was evaluated 1 to 3 months later. The target values of TSAT and serum ferritin were 20 ~ 50% and 100 ~ 500 μg /L, respectively. If the values of TSAT and serum ferritin were not up to the target level, we could treat the patients with intravenous iron. Patients with serum ferritin ≥500 μg/L should not receive iron supplements. Hb levels were monitored at baseline, 2 weeks, 1 month, 2 months, 3 months, 6 months, 9 months, 12 months, 15 months, 18 months and 24 months; blood pressure and cardiovascular parameters were monitored at baseline and after follow-up, and we observed the occurrence and prognosis of cardiovascular and cerebrovascular complications after follow-up.

### Measurements and variable definitions

Measurements: (1) Clinical data included age, sex, weight, height, duration of dialysis, blood pressure, diabetes, medication history, PD modalities, and urine volume, (2) Biochemical data and cardiovascular parameters included serum, dialysate and urine urea nitrogen, creatinine and glucose, serum albumin, Hb, total cholesterol, triglycerides, low-density lipoprotein cholesterol (LDL-C), high-density lipoprotein cholesterol (HDL-C), ferritin, serum iron, total iron-binding capacity, hypersensitive C-reactive protein (CRP), fasting blood glucose, N-terminal pro-brain natriuretic peptide (NT-proBNP), troponin T (TnT), troponin I (TnI) and interventricular septal (IVS), left ventricular posterior wall thickness (LVPW), cardiothoracic ratio (CTR), and left ventricular ejection fraction (LVEF). Laboratory tests were performed at each follow-up visit, echocardiography was performed annually, (3) Cardio-cerebrovascular complications included angina pectoris, myocardial infarction and other coronary heart diseases, heart failure caused by various reasons, atrial fibrillation, atrioventricular block and other arrhythmias, stroke (cerebral infarction, cerebral hemorrhage) which were recorded by diagnosis codes, laboratory and image studies, and (4) The changes in the abovementioned parameters were recorded at baseline and after treatment.

Variable definitions: Renal anemia is defined as anemia caused by impaired production of EPO from the kidney and a hemoglobin level of <110 g/L. The control rate of Hb means the proportion of Hb controlled between 110 and 130 g/L. Mean arterial pressure (MAP) is defined as diastolic blood pressure + 1/3 (systolic blood pressure - diastolic blood pressure), systolic blood pressure and diastolic blood pressure were conducted by office, home or ambulatory blood pressure monitoring and we chose the mean value of blood pressure over multiple time points; nocturnal hypertension is defined as a blood pressure value of ≥120/70 mmHg at night (10) which was measured by home and ambulatory blood pressure monitoring; initial dialysis was defined as dialysis time within 3 months at the time of enrolment; TSAT was defined as serum iron/total iron-binding capacity×100%; CTR was defined as cardiac diameter/thoracic diameter in chest X-ray.

### Statistical analysis

We used the propensity score matching method to improve the equilibrium between the two groups. Statistical analysis was performed using SPSS statistical software version 26.0 (SPSS Inc., Chicago, IL, United States). Descriptive parametric data consistent with a normal distribution are expressed as the mean ± standard deviation (SD), and nonparametric data are expressed as the median (interquartile range). We used Student’s t-test and Mann–Whitney tests to compare continuous variables between the two groups. Categorical variables were expressed as percentages and compared using the *χ*2-test. The univariate Cox regression model was used to calculate the hazard ratio (HR) and 95% confidence interval (CI) of each factor. GraphPad 8.0 software was used to draw the survival curve of cardio-cerebrovascular complications by Kaplan–Meier. The log-rank test was used to compare the survival curve between the two groups. *p* < 0.05 was considered to indicate statistical significance.

## Results

### Patient demographics and baseline characteristics

A total of 120 PD patients with renal anemia were included, i.e., 60 PD patients in roxadustat group and 60 PD patients in rHuEPO group. There were no significant differences in clinical and biochemical data at baseline between the two groups of PD patients (*p* > 0.05) (Table 1).

**Table 1:** Baseline characteristics of PD patients in the two groups.

	Roxadustat (*n* = 60)	rHuEPO (*n* = 60)	*p-*value
Age (years)	46.6 ± 10.8	49.3 ± 11.2	0.172
Male, *n* (*%*)	29 (48.3)	29 (48.3)	1.000
Weight (kg)	58.2 ± 9.9	59.7 ± 10.8	0.411
Duration of dialysis (months)	6.5 (0–23.5)	9.0 (0–24.8)	0.386
MAP (mmHg)	108.1 ± 11.8	107.5 ± 11.7	0.757
Hypertension, *n* (*%*)	55 (91.6)	54 (90)	1.000
Diabetes, *n* (*%*)	6 (10)	7 (11.7)	1.000
rHuEPO use before, *n* (*%*)	27 (45)	29 (55.8)	0.855
Iron treatment, *n* (*%*)	50 (83.3)	52 (86.7)	0.799
Initial dialysis, *n* (*%*)	27 (45)	25 (41.7)	0.854
PD modalities, *n* (*%*)			
CAPD	5 (8.3)	3 (5)	0.717
DAPD	51 (85)	55 (91.7)	0.394
APD	4 (6.7)	2 (3.3)	0.678
KT/V	1.86 ± 0.5	1.97 ± 0.5	0.217
Ccr (L/1.73m^2^)	68.6 ± 27.1	77.6 ± 29.5	0.447
Hb (g/L)	89.6 ± 13.7	90.2 ± 13.5	0.825
Total cholesterol (mmol/L)	4.5 ± 1.1	4.6 ± 1.1	0.517
Triglycerides (mmol/L)	1.7 ± 0.9	1.8 ± 1.0	0.901
HDL-C (mmol/L)	1.1 ± 0.3	1.2 ± 0.4	0.267
LDL-C (mmol/L)	2.5 ± 0.7	2.5 ± 0.8	0.830
Albumin (g/L)	37.6 ± 4.4	38.7 ± 4.6	0.189
Iron metabolism			
Ferritin ≥100 μg/L and TSAT ≥20%,*n* (*%*)	31 (51.7)	29 (48.3)	0.855
Ferritin <100 μg/L or TSAT <20%, *n* (*%*)	29 (48.3)	31 (51.7)	0.855
hs-CRP (mg/L)	1.1 (0.5–2.2)	1.1 (0.5–2.3)	0.780
Fasting blood glucose (mmol/L)	5.04 ± 0.67	5.14 ± 0.83	0.433

MAP, mean arterial pressure; CAPD, continuous ambulatory peritoneal dialysis; DAPD, daytime ambulatory peritoneal dialysis; APD, automated peritoneal dialysis; Kt/V, weekly urea clearance index; Ccr, weekly creatinine clearance; Hb, hemoglobin; HDL-C, high-density lipoprotein cholesterol; LDL-C, low-density lipoprotein cholesterol; TSAT, transferrin saturation; hs-CRP, high-sensitivity C-reactive protein.

### Hb levels

**Figure 1:**
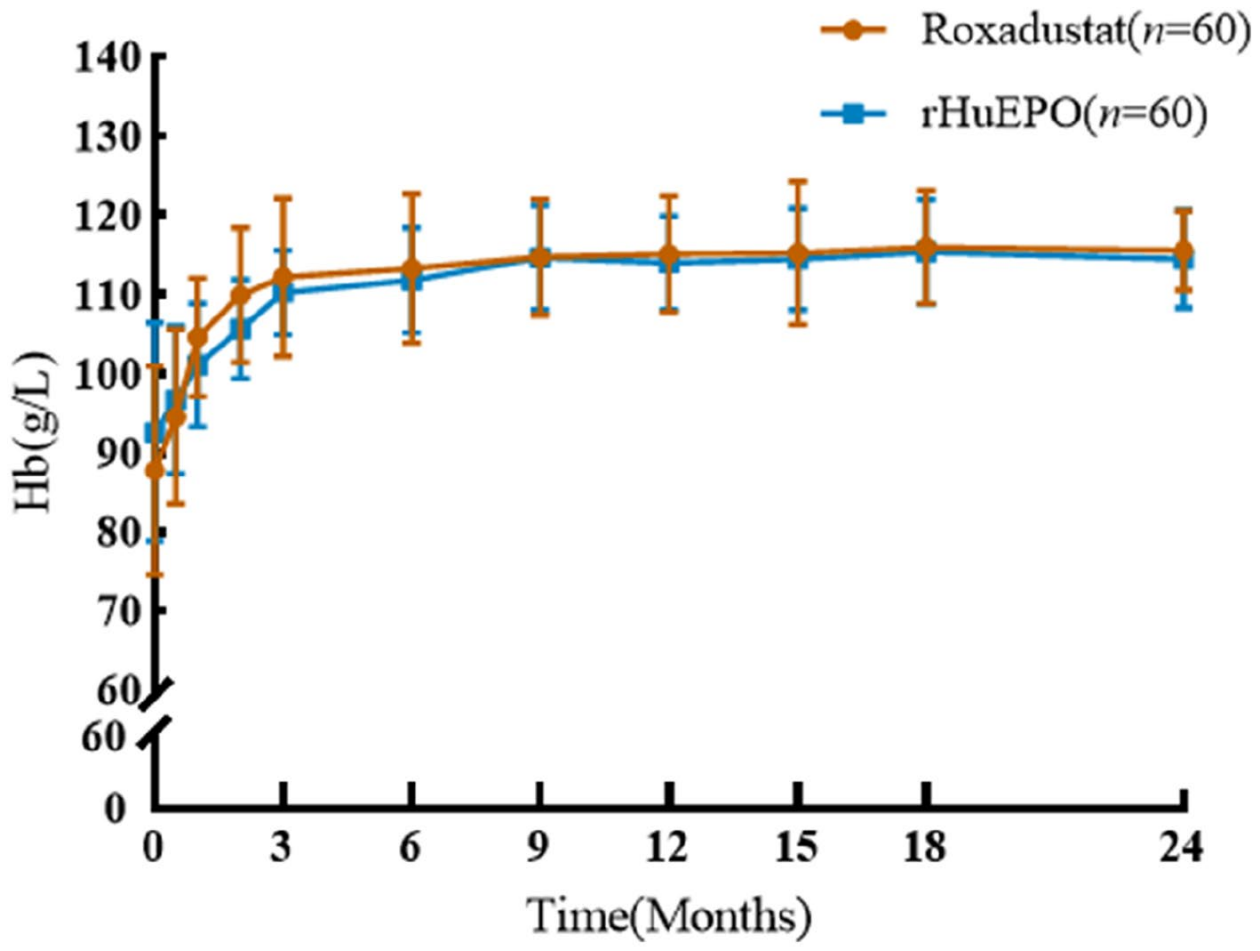
Comparison of Hb before and after treatment between the two groups.

There was no significant difference in Hb between the two groups of PD patients at baseline, and both Hb levels increased significantly after treatment (Figure 1). There was no significant difference in Hb between the two groups at the 24-month follow-up (115.1 ± 5.5 vs. 114.8 ± 6.0, *p* = 0.716). The control rate of Hb in roxadustat group and rHuEPO group were 90 and 88.3%, respectively (*p* > 0. 05).

### Blood pressure

There were no significant differences in blood pressure between the two groups at baseline, and there were no significant changes in systolic blood pressure, diastolic blood pressure or nocturnal hypertension in roxadustat group before and after treatment (*p* > 0.05). After 24 months of follow-up, both systolic blood pressure and diastolic blood pressure in rHuEPO group were significantly higher than those at baseline, and the incidence of nocturnal hypertension increased (*p* < 0.05). However, after 24 months of follow-up, systolic and diastolic blood pressure and nocturnal hypertension increased in rHuEPO group compared with roxadustat group (*p* < 0.05) (Table 2).

**Table 2:** Comparison of blood pressure, cardiovascular parameters between the two groups of PD patients at baseline and after 24 months of follow-up.

	Roxadustat (*n* = 60)	rHuEPO (*n* = 60)
	Baseline variable	After 24 months of follow-up	Change from baseline	Baseline	After 24 months of follow-up	Change from baseline
Systolic blood pressure (mmHg)	131.3 ± 15.9	130.0 ± 12.5	−1.28 ± 13.4	131.1 ± 15.9	138.1 ± 14.0^*#^	7.02 ± 15.1^#^
Diastolic blood pressure (mmHg)	85.0 ± 10.2	83.1 ± 7.7	−1.93 ± 9.0	83.8 ± 10.1	89.8 ± 9.4^*#^	5.35 ± 10.8^#^
Nocturnal hypertension, *n* (*%*)	8 (13.3)	5 (8.3)	−3 (−5)	7 (11.7)	18 (30)^*#^	11 (18.3)^#^
Urine volume (mL)	1,100 (112–1457.5)	1,075 (157.5, 1437.5)	−50 (−100, 0)	1,100 (725–1337.5)	800 (450, 1087.5)^*#^	−200 (−487, −50)^#^
Weight (kg)	59.4 ± 9.9	57.8 ± 10.4	−0.4 ± 4.4	60.2 ± 10.8	59.7 ± 11.9	−0.0 ± 6.8
NT-proBNP (pmol/L)	170.2 (56.5, 475.8)	114.3 (34.4, 300.5)	−5.4 (−167.2, 158.5)	119.1 (60.1, 456.3)	424.4 (155.5, 1350.5)^*#^	95.3 (−17.2, 494.0)^#^
TnT (ng/mL)	0.019 (0.014, 0.034)	0.027 (0.021, 0.064)	0.007 (−0.004, 0.019)	0.021 (0.0125, 0.036)	0.066 (0.047, 0.0995)^*#^	0.048 (0.029, 0.072)^#^
TnI (ng/mL)	0.03 (0.02, 0.03)	0.0275 (0.015, 0.03)	0.005 (0,0.015)	0.035 (0.025, 0.04)	0.05 (0.0395, 0.06)^*#^	0.02 (0.01, 0.04)^#^
IVS (mm)	9.94 ± 1.52	10.03 ± 1.75	−0.09 ± 1.74	10.26 ± 1.92	10.52 ± 1.53	0.28 ± 1.82
LVPW (mm)	9.94 ± 1.23	10.06 ± 1.85	−0.38 ± 1.40	9.78 ± 1.43	10.1 ± 1.42	0.26 ± 1.94
LVEF (%)	62.8 ± 4.5	63.2 ± 4.8	0.9 ± 6.7	63.8 ± 6.1	60.9 ± 4.7^*#^	−3.3 ± 7.1^#^
Cardiothoracic ratio	0.48 ± 0.06	0.49 ± 0.04	−0.02 ± 0.05	0.48 ± 0.05	0.51 ± 0.07^*^	0.04 ± 0.06^#^

NT-proBNP, N-terminal pro-brain natriuretic peptide; TnT, troponin T; TnI, troponin I; IVS, interventricular septal; LVPW, left ventricular posterior wall thickness; LVEF, left ventricular ejection fraction.

Compared with baseline, ^*^p<0.05; Compared with roxadustat group, ^#^*p*<0.05.

### Cardiovascular parameters and cardio-cerebrovascular complications

**Figure 2:**
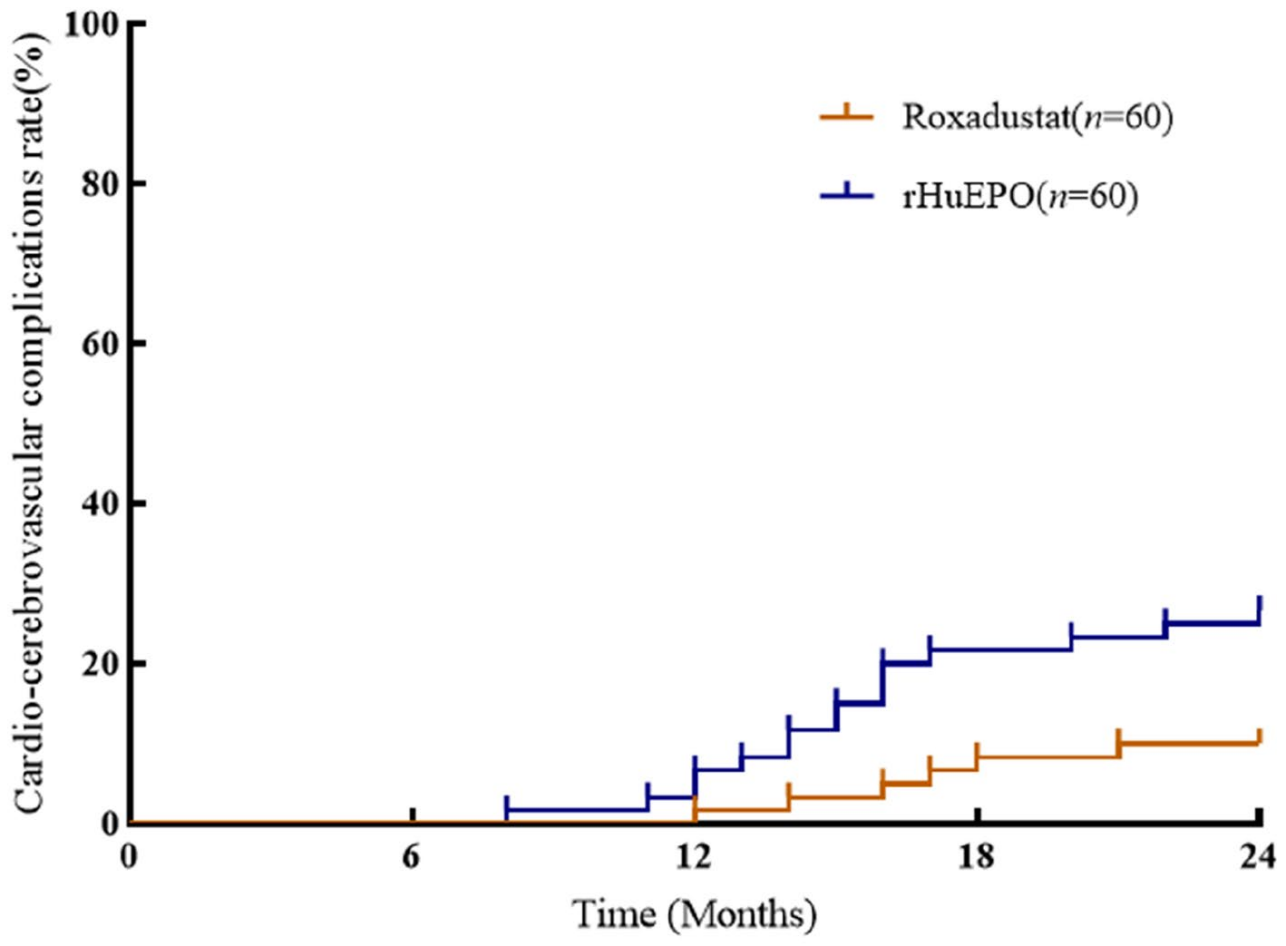
Comparison of incidence of cardio-cerebrovascular complications between the two groups. There was a significant difference among the two groups after 24  months of follow-up (*p* < 0.05).

There were no significant differences in cardiovascular parameters before and after treatment in roxadustat group (*p* > 0.05). NT-proBNP, TnT, TnI and CTR increased and LVEF decreased in rHuEPO group compared with baseline (*p* < 0.05), but there was no significant change in IVS and LVPW (*p* > 0.05). Compared with roxadustat group, the levels of NT-proBNP, TnT, TnI and CTR in rHuEPO group increased, while LVEF decreased (*p* < 0.05). IVS and LVPW showed no significant difference (*p* > 0.05) (Table 2). Compared with baseline, rHuEPO group had more cardiovascular and cerebrovascular complications after treatment (26.7% vs. 0, *p* < 0.05). Cardiovascular and cerebrovascular complications increased in the rHuEPO group compared with those in roxadustat group after follow-up (26.7% vs. 10%, *p* < 0.05) (Table 3, Figure 2).

**Table 3:** Comparison of cardio-cerebrovascular complications and prognosis of PD patients in the two groups during 24  months of follow-up.

Variable	HR	95% CI	*p*
Age (years)	1.045	1.006–1.086	0.022
Hb (g/L)	1.002	0.971–1.034	0.889
Systolic blood pressure (mmHg)	1.036	1.009–1.065	0.009
Diastolic blood pressure (mmHg)	1.033	0.989–1.079	0.139
Fasting blood glucose (mmol/L)	2.532	1.593–4.024	<0.001
rHuEPO use before	2.620	1.068–6.429	0.035
Roxadustat treatment	0.338	0.132–0.864	0.023
KT/V	0.619	0.233–1.641	0.335
Ccr (L/1.73 m^2^)	0.987	0.972–1.003	0.110
Total cholesterol (mmol/L)	1.151	0.775–1.709	0.485
LDL-C (mmol/L)	1.442	0.736–2.825	0.286
Blood albumin (g/L)	0.942	0.866–1.025	0.167
Hypersensitive CRP (mg/L)	0.950	0.834–1.081	0.436

HR, hazard ratio; CI, confidence interval; Hb, hemoglobin; Kt/V, weekly urea clearance index; Ccr, weekly creatinine clearance; LDL-C, low-density lipoprotein cholesterol; Hypersensitive CRP, hypersensitive C-reactive protein.

### Risk factors for cardio-cerebrovascular complications

Cox regression analysis showed that age, systolic blood pressure, fasting blood glucose, and rHuEPO use before baseline were risk factors for cardio-cerebrovascular complications in PD patients, while treatment with roxadustat was a protective factor for cardiovascular and cerebrovascular complications (Table 4).

**Table 4:** Analysis of risk factors for cardiovascular and cerebrovascular complications in PD patients.

	Roxadustat (*n* = 60)	rHuEPO (*n* = 60)
Cardio-cerebrovascular complications, *n* (*%*)	6 (10)	16 (26.7)^*^
Coronary heart diseases, *n* (*%*)	1 (1.7)	5 (8.3)
Heart failure, *n* (*%*)	3 (5)	6 (10)
Arrhythmia, *n* (*%*)	0	1 (2)
Stroke, *n* (*%*)	2 (3.3)	4 (6.7)
Changed to HD or combined with HD due to cardio-cerebrovascular complication, *n* (*%*)	2 (3.3)	5 (8.3)
All-cause mortality, *n* (*%*)	0	1 (1.7)

HD, Hemodialysis; compared with roxadustat group, *p<0.05.

## Discussion

Renal anemia is a common complication in patients with CKD and is associated with increased cardiovascular events, hospitalization rates and mortality (11). Roxadustat is an oral HIF-PHI that can stimulate the production of endogenous erythropoietin (EPO) in the human body, and the process is very similar to the physiological regulation process of hypoxia at high altitude. HIF-PHI can increase the sensitivity of EPO receptors and promote iron absorption, which is more conducive to its clinical effect of improving renal anemia (12). Li et al. (13) showed that the effect of HIF-PHI in improving anemia was not affected by the inflammatory state and iron concentration in the human body. However, there have been few studies on the cardiovascular parameters and cardio-cerebrovascular complications of roxadustat. Therefore, this study aimed to observe cardiovascular parameters and cardio-cerebrovascular complications in PD patients to deepen the clinical understanding.

ESAs are direct vasoconstrictors that can raise blood pressure, and can also raise blood pressure by stimulating the production of endothelin, epinephrine, renin, and angiotensin. Furthermore, the effect of ESAs on blood viscosity is also a mechanism of hypertension (14); as a result, after using rHuEPO in clinical practice, PD patients are more likely to develop hypertension. In this study, compared with ESAs, roxadustat can correct anemia without increasing cardiovascular and cerebrovascular burden in PD patients. The lower blood pressure of roxadustat group has a protective effect on cardiovascular and cerebrovascular diseases. These results are related to the mechanism of HIF-PHI. Yu et al. (15) showed that HIF-PHI could reduce hypertension associated with high renin-angiotensin system activity or endothelial nitric oxide synthase (eNOS) deficiency in the angiotensin II (Ang II) hypertensive mouse model. Roxadustat downregulated the expression of angiotensin receptor 1, increased the expression of angiotensin receptor 2, eNOS and HIF1α protein levels, prevented Ang II-induced oxidative stress, eliminated hypertensive response, and prevented vascular thickening, myocardial hypertrophy and renal injury. HIF-PHI also regulated angiogenesis, glucose metabolism, cell proliferation and apoptosis in an animal model of nephropathy with metabolic syndrome. HIF-PHI increased renal glucose excretion, reduced fat weight, and corrected hypertension. The mechanism of HIF-PHI for lowering blood pressure could be that it induces the transcription of vasodilation-related genes (16). In this study, compared with rHuEPO group, the blood pressure of PD patients in roxadustat group was significantly lower, and the abovementioned mechanisms provide a certain theoretical basis for this result.

Signore et al. (16) showed that the level of NT-proBNP decreased, left ventricular end-diastolic pressure decreased, LVEF increased, cardiac systolic and diastolic function improved, cardiac blood flow and myocardial oxygenation increased, and myocardial hypertrophy, fibrosis, and remodeling decreased in renal anemia rats treated with HIF-PHI. Animal model analysis indicated that HIF-1 plays a key protective role in the pathophysiological mechanism of ischemic heart disease and pressure overload heart failure (17). HIF-1 is a transcription factor that acts as a major regulator of oxygen homeostasis, it regulates angiogenesis and vascular remodeling, and utilizes oxygen by regulating glucose metabolism and redox homeostasis. In this study, PD patients treated with roxadustat had lower blood pressure and better cardiovascular parameters than those treated with rHuEPO, these results are considered to be related to the mechanism mentioned above.

Provenzano et al. (18) showed that roxadustat did not increase the risk of major adverse cardiovascular events (MACE, including cardiovascular death, myocardial infarction and stroke), MACE+ (MACE, unstable angina and congestive heart failure requiring hospitalization), or all-cause death compared with placebo in 4277 non-dialysis patients with CKD renal anemia. Recent randomized phase 3 trial study compared dialysis patients treated with roxadustat versus rHuEPO, there were no statistically significant differences in cardiovascular events and adverse events between the two groups (19). Barratt et al. (20) found that there were no significant differences in the risk of major adverse cardiovascular events (MACE, including cardiovascular death, myocardial infarction and stroke) and MACE+ (MACE, unstable angina and congestive heart failure requiring hospitalization) in non-dialysed CKD patients with renal anemia when the patients were treated with roxadustat compared with rHuEPO, but subgroup analysis found that roxadustat group had lower rates of hospitalization for congestive heart failure or unstable angina. Provenzano et al. (21) showed that roxadustat reduced the risk of MACE by 30% and MACE+ by 34% in initial dialysis patients compared with rHuEPO, therefore, in the study, roxadustat had a lower risk of MACE/MACE+ compared with rHuEPO in patients new to dialysis. Our study is the first time to compare blood pressure, the cardiovascular parameters and cardio-cerebrovascular complications treated with roxadustat versus rHuEPO only in PD patients. We had a continuous normative dynamic observation on cardio-cerebrovascular parameters and events. The highlight of this study is that roxadustat had a cardio-cerebrovascular protective effect on PD patients. The possible mechanism is that HIF and its family of proteins regulate an oxygen-dependent signalling cascade, perceive and regulate the hypoxia and ischemia of cardio-cerebrovascular vessels and play a protective role in regulation. In addition, HIF acts on endothelial cells, vascular smooth muscle cells and macrophages, and plays a key protective role in cardio-cerebrovascular atherosclerotic diseases through cell-specific responses (22, 23). Recently, Zhao et al. (24) found that roxadustat in routine treatment doses did not affect platelet production, activation or thrombosis and had significant cardiovascular and cerebrovascular protection advantages compared with ESAs. The cardiovascular protective effect of roxadustat could be observed in the above animal experiments and clinical investigations.

Acet et al. (25) showed that elevated blood glucose was an independent predictor of MACE, and Monami et al. (26) showed that the risk of MACE reduced and renal adverse events correspondingly reduced after blood glucose control. In this study, elevated fasting blood glucose was a risk factor for cardio-cerebrovascular complications in PD patients, suggesting that active control of blood glucose in PD patients is needed to reduce the incidence of cardio-cerebrovascular complications.

However, there are some limitations in this study. It is a retrospective study in a single center. And then the 2 years of follow-up time of cardio-cerebrovascular complications in this study was insufficient. Therefore, the results of this study need to be further validated with longer follow up in a multi-center prospective study.

## Conclusion

In this study, compared with rHuEPO in PD patients, roxadustat has less impact on blood pressure and cardiovascular parameters, and the incidence of cardio-cerebrovascular complications is lower. Treatment with roxadustat in PD renal anemia patients offers cardio-cerebrovascular protection, especially in PD patients with cardiovascular complications or elevated cardiovascular parameters, which is worthy of in-depth clinical study and discussion. It is necessary to further increase the sample size and observation parameters to make the research results more objective and comprehensive.

## Data availability statement

The original contributions presented in the study are included in the article/supplementary material, further inquiries can be directed to the corresponding author.

## Ethics statement

The studies involving human participants were reviewed and approved by the Local Ethics Committee of Jinling Hospital. Written informed consent to participate in this study was provided by the participants' legal guardian/next of kin.

## Author contributions

SC, TZ, and LY conceived the study, researched literatures and develop the protocol. YY and SC obtained ethical approval. LY and ZZ performed data analysis. SC, TZ, and LY wrote the first draft of the manuscript. YY approved the final version of this manuscript. All authors contributed to the article and approved the submitted version.

## Funding

This study was financially supported by the Jiangsu Clinical Medical Center Project (Grant no. YYS2016001).

## Conflict of interest

The authors declare that the research was conducted in the absence of any commercial or financial relationships that could be construed as a potential conflict of interest.

## Publisher’s note

All claims expressed in this article are solely those of the authors and do not necessarily represent those of their affiliated organizations, or those of the publisher, the editors and the reviewers. Any product that may be evaluated in this article, or claim that may be made by its manufacturer, is not guaranteed or endorsed by the publisher.
